# Evaluation of the *IL2/IL21*, *IL2RA* and *IL2RB* genetic variants influence on the endogenous non-anterior uveitis genetic predisposition

**DOI:** 10.1186/1471-2350-14-52

**Published:** 2013-05-15

**Authors:** María Carmen Cénit, Ana Márquez, Miguel Cordero-Coma, Alejandro Fonollosa, Alfredo Adán, Agustín Martínez-Berriotxoa, Victor Llorenç, David Díaz Valle, Ricardo Blanco, Joaquín Cañal, Manuel Díaz-Llopis, José Luis García Serrano, Enrique de Ramón, María José del Rio, Marina Begoña Gorroño- Echebarría, José Manuel Martín-Villa, Norberto Ortego-Centeno, Javier Martín

**Affiliations:** 1 Instituto de Parasitología y Biomedicina López-Neyra, IPBLN, CSIC, Parque Tecnológico Ciencias de la Salud, Avenida del Conocimiento s/n 18100-Armilla, Granada, Spain; 2 Ophthalmology Department, Hospital de León, León, Spain; 3 Internal Medicine Department, Hospital de Cruces, Bilbao, Spain; 4 Ophthalmology Department, Hospital Clínic, Barcelona, Spain; 5 Ophthalmology Department, Hospital Clínico San Carlos, Madrid, Spain; 6 Rheumatology Department, Hospital Marqués de Valdecilla, IFIMAV, Santander, Spain; 7 Ophthalmology Department, Hospital Marqués de Valdecilla, Santander, Spain; 8 Ophthalmology Department, Hospital Universitario La Fe, Valencia, Spain; 9 Ophthalmology Department, Hospital Clínico San Cecilio, Granada, Spain; 10 Internal Medicine Department, Hospital Carlos Haya, Málaga, Spain; 11 Ophthalmology Department, Hospital Carlos Haya, Málaga, Spain; 12 Ophthalmology Department, Hospital Universitario Principe de Asturias, Alcalá de Henares, Spain; 13 Immunology Department, Facultad de Medicina, Universidad Complutense de Madrid, Madrid, Spain; 14 Internal Medicine Department, Hospital Clínico San Cecilio, Granada, Spain

**Keywords:** Uveitis, *IL2*, *IL21*, *IL2RA*, *IL2RB*, Polymorphisms, Association study, Genetic susceptibility

## Abstract

**Background:**

Recently, different genetic variants located within the *IL2/IL21* genetic region as well as within both *IL2RA* and *IL2RB* loci have been associated to multiple autoimmune disorders. We aimed to investigate for the first time the potential influence of the *IL2/IL21, IL2RA* and *IL2RB* most associated polymorphisms with autoimmunity on the endogenous non-anterior uveitis genetic predisposition.

**Methods:**

A total of 196 patients with endogenous non-anterior uveitis and 760 healthy controls, all of them from Caucasian population, were included in the current study. The *IL2/IL21* (rs2069762, rs6822844 and rs907715), *IL2RA* (2104286, rs11594656 and rs12722495) *and IL2RB* (rs743777) genetic variants were genotyped using TaqMan® allelic discrimination assays.

**Results:**

A statistically significant difference was found for the rs6822844 (*IL2/IL21* region) minor allele frequency in the group of uveitis patients compared with controls (*P-*_value_=0.02, OR=0.64 CI 95%=0.43-0.94) although the significance was lost after multiple testing correction. Furthermore, no evidence of association with uveitis was detected for the analyzed genetic variants of the *IL2RA* or *IL2RB* loci.

**Conclusion:**

Our results indicate that analyzed *IL2/IL21*, *IL2RA and IL2RB* polymorphisms do not seem to play a significant role on the non-anterior uveitis genetic predisposition although further studies are needed in order to clear up the influence of these loci on the non-anterior uveitis susceptibility.

## Background

Uveitis is an intraocular inflammatory disorder mediated by a wide range of causes, including exogenous and endogenous agents. This condition is considered a major source of visual impairment being the fourth cause of blindness worldwide [1,2]. Endogenous uveitis is an inflammatory response mediated by immune system driven for a loss of tolerance against ocular antigens [3].

A shared genetic component among different autoimmune diseases is well established. This fact has suggested that these disorders may be influenced by both disease-specific and common molecular mechanisms [4]. Indeed, the autoimmune uveitis also seems to share common genetic factors with other autoimmune disorders [5]. To date, different studies have been conducted in order to identify the uveitis genetic component [6-9], although results have been inconclusive. It is important to note that the majority of studies on uveitis susceptibility have been focused on anterior uveitis and few studies have been carried out to determine the non-anterior uveitis genetic background.

Interleukin 2 (IL-2) was initially identified as an autocrine product from activated T cells, although later was found that IL-2 plays a crucial role in the maintenance of immune system homeostasis and self-tolerance [10]. The main non-redundant function of this cytokine seems to be the regulation of peripheral tolerance by supporting the survival and function of regulatory T cells. In fact, mice deficient in IL-2, IL-2Rα, and IL-2Rβ exhibit autoimmunity [11,12]. In uveitis condition, IL-2 induces the expansion of Th17 cells, which were found to be elevated in uveitis patients [13]. Moreover, Daclizumab therapy, a monoclonal antibody against alpha subunit of the IL-2 receptor, reduces the active uveitis inflammation [14].

On the other hand, interleukin 21 (IL-21) is a potent immunomodulatory cytokine with pleiotropic effects on both innate and adaptive immune responses. IL-21 is produced mainly by CD4+ T cells and promotes the effector CD8+ T cells and NK cells function and expansion. In addition, it is also critical for B-cell differentiation into plasma cells and negatively regulates the function of dendritic cells [15]. Recently, it has been suggested that the dendritic cells maduration seems play a role in the pathogenesis of endogenous uveitis [16]. Furthermore, IL-21 seems to be involved in the Behçet’s disease (BD) [17] as well as in the Vogt-Koyanagi-Harada (VKH) syndrome pathogenesis [18], both disorders strongly characterized by the presence of uveitis condition, probably by promoting IL-17 secretion.

Different studies with animal models have clearly supported the involvement of IL-21 as well as IL2/IL2R pathway as potential drivers of autoimmunity [19-24]. Additionally, genetic associations between different polymorphisms located within the *IL2/IL21* region as well as within both *IL2RA* and *IL2RB* loci and several autoimmune diseases have also been reported [25-34].

Regarding the *IL2RA* gene, different independent signals on this gene were identified by fine mapping of the region, and their minor alleles were related to inherited lower circulating levels of the soluble IL-2RA protein, [25-27,29]. IL-2RA is expressed constitutively on regulatory T cells, a population of T cells that has been shown to have a potent ability to suppress autoreactive T cells [35].

Taking into account all of the above, we herein aimed to investigate whether the previously most associated polymorphisms with different autoimmune disorders located in *the IL2/IL21, IL2RA* and *IL2RB loci* are involved in the genetic predisposition to autoimmune uveitis. For this purpose, we studied three SNPs located within the *IL2/IL21* region (rs907715, located within the *IL21* gene and most associated SNP with SLE in a recent fine mapping, rs2069762 situated within the *IL2* gene and rs6822844 positioned in the *IL2/IL21* inter-genic region), three functional independent Single Nucleotide Polymorphisms (SNPs) related with lower circulating levels of the soluble IL-2RA (rs11594656, rs2104286 and rs12722495) and one SNP in the *IL2RB* locus (rs743777), all of them previously associated to multiple autoimmune conditions through genome-wide association studies [28,30-34].

## Methods

### Study population

We included a total of 196 patients with endogenous uveitis, excluding the anterior uveitis forms as well as uveitis associated with systemic immune-mediated diseases except Vogt-Koyanagi-Harada syndrome, and 760 ethnically matched healthy controls, all of them from Caucasian population. Furthermore, uveitis patients were classified depending on the part of eye affected by the inflammatory process in intermediate uveitis (IU), posterior uveitis (PU) or panuveitis (PAN) [36]. Written informed consent and approval of the local ethical committees were obtained and the research followed the tenets of the Declaration of Helsinki. The design of the work was approved by the Ethics Committee of Granada (Spain). The Ethics Committees of the Hospital de León (León), Hospital Universitario Príncipe de Asturias (Alcalá de Henares), Hospital de Cruces (Bilbao), Hospital Clinic (Barcelona), Hospital Clínico San Carlos (Madrid), Hospital Marqués de Valdecilla (Santander), Hospital Universitario La Fe (Valencia), Hospital Clínico San Cecilio (Granada) and Hospital Carlos Haya (Málaga) also approved the study.

### Genotyping and SNP selection

Genomic DNA was extracted from peripheral white blood cells following standard procedures. The single-nucleotide polymorphisms (SNPs) were genotyped using pre-designed TaqMan® allelic discrimination assays in a 7900HT Real-Time polymerase chain reaction (PCR) System from Applied Biosystems (Foster City, CA, USA).

We carried out the genotyping of the rs2069762, rs6822844 and rs907715 genetic variants, located within the 4q27 (*IL2/IL21*) region, rs11594656, rs2104286 and rs12722495 in the *IL2RA* gene and rs743777 located within the *IL2RB* locus.

### Statistical analysis

The Linux software Plink v1.7 (http://pngu.mgh.harvard.edu/purcell/plink/) was used to perform 2x2 contingency tables, χ2 and/or Fisher’s exact tests, when appropriate. The genotype, allele and carrier frequencies were compared between patients, subgroup of patients and controls. Odds ratios (OR) and 95% confidence intervals (CI) were obtained according to Woolf’s method. Hardy–Weinberg equilibrium (HWE) was tested for all SNPs at significance level=0.01. Since different polymorphisms were analyzed in the current study, *the* Benjamini & Hochberg (1995) step-up false discovery rate (FDR) control correction for multiple testing was applied to the *P*-values. *P*-values after FDR correction lower than 0.05 were considered as statistically significant.

Furthermore, the effect of the analyzed genetic variants located within the 4q27 as well as within the *IL2RA* gene was analyzed either in isolation or by allelic combination analysis in order to cover a higher variability of these chromosome regions.

The overall statistical power of our study according to Power Calculator for Genetic Studies 2006 software (http://www.sph.umich.edu/csg/abecasis/CaTS/), is shown in online Additional file 1: Table S1.

## Results

Table 1 describes the main clinical and demographic characteristic of uveitis patients included in the current study and Table 2 shows the genotypic and allelic frequencies in cases and controls. The intraocular inflammation seen in patients included intermediate uveitis (18.4%), posterior uveitis (55.1%), and panuveitis (23.5%).

**Table 1:** Clinical and demographic features of uveitis patients and controls

**General characteristics**	**Uveitis patients (n=196)**	**Controls (n=760)**
Male(%)/Female (%)	83 (42.3)/113 (52.1)	**254 (33.4)/506 (66.6)**
Age (mean ± SD)	46.3 ±15.48	**45.12 ± 12.21**
Intermediate Uveitis (%)	36 (18.4)	-
Posterior Uveitis (%)	108 (55.1)	-
Panuveitis (%)	46 (23.5)	-
Bilateral affection (%)	149 (68.7)	-
Vitritis (%)	135 (62.2)	-
Macular edema (%)	89 (41.0)	-
Retinal vasculitis (%)	83 (38.2)	-
Choroidal neovascularization (%)	20 (9.2)	-

**Table 2:** **Allelic and genotype frequencies of *IL2/IL21, IL2RA *and *IL2RB *genetic variants in uveitis patients and healthy controls from Spanish population**

**SNP**	**1/2**	**Subgroup (n)**	**11**	**12**	**22**	**MAF (%)**	**Allelic *P*-value**	**OR (95% CI)**
***IL2/IL21***								
rs2069762	**C/A**	Controls (n=760)	61 (8.03)	315 (41.45)	384 (50.53)	437 (28.75)		
		Uveitis (n=195)	17 (8.71)	85 (43.60)	93 (47.69)	119 (30.51)	0.49	1.09 (0.85-1.40)
rs6822844	**T/G**	Controls (n=760)	15 (1.97)	156 (20.53)	589 (77.50)	186 (12.24)		
		Uveitis (n=196)	2 (1.02)	28 (14.29)	166 (84.69)	32 (8.16)	0.02*	0.64 (0.43-0.94)
rs907715	**T/C**	Controls (n=760)	81 (10.66)	330(43.42)	349(46.92)	492 (32.37)		
		Uveitis (n=196)	26 (13.27)	70 (35.71)	100 (51.02)	122 (31.12)	0.64	0.94 (0.74-1.20)
***IL2RA***								
rs12722495	**G/A**	Controls (n=760)	3 (0.39)	114 (15.00)	643 (84.61)	120 (7.90)		
		Uveitis (n=196)	2 (1.02)	29 (14.80)	165 (84.18)	33 (8.42)	0.73	1.07 (0.72-1.60)
rs2104286	**C/T**	Controls (n=760)	19 (2.50)	270 (35.53)	471 (61.97)	308 (20.26)		
		Uveitis (n=196)	10 (5.10)	60(30.61)	126 (64.29)	80 (20.41)	0.95	1.01 (0.77-1.33)
rs11594656	**T/A**	Controls (n=760)	84 (11.05)	346(45.53)	330(43.42)	514 (33.82)		
		Uveitis (n=196)	23 (11.79)	69 (35.38)	103 (52.82)	115 (29.49)	0.10	0.82 (0.64-1.04)
***IL2RB***								
rs743777	**G/A**	Controls (n=760)	86(11.32)	349(45.92)	325(42.76)	521 (34.28)		
		Uveitis (n=195)	26 (13.40)	72 (37.11)	96 (49.48)	124 (31.96)	0.39	0.90 (0.71-1.14)

*P_FDR-corrected_=0.17.

SNP; Single Nucleotide Polymorphism.

MAF; Minor Allele Frequency.

OR; Odds Ratio.

No statistically significant deviation from Hardy-Weinberg equilibrium (P≤0.01) was observed for all the studied SNPs in the control set and the frequencies of the analyzed SNPs were in agreement with the data of the HapMap project. Genotyping success rate was higher than 95% for all analyzed SNPs. In addition, randomly selected samples were genotyped twice to verify the genotyping accuracy and 99% of the genotypes were identical.

In the case/control analysis, we detected a decrease of the minor allele frequency of the rs6822844 (*IL2/IL21* region) in the group of uveitis patients (*P-*_value_=0.02, OR=0.64 CI 95%=0.43-0.94). Nevertheless, when we applied the FDR correction the significance was lost (*P*_FDR_=0.17) (Table 2). Additionally, no statistical significant differences were observed in the genotype, allele and carriers frequencies for the analyzed *IIL2RA* and *IL2RB* SNPs between uveitis patients and controls.

When we compared the different subgroups of uveitis patients stratified according to the different main features showed in Table 1 no statistically significant differences were detected for none of the analyzed polymorphisms (data not shown).

On the other hand, the allelic combination analysis of the studied polymorphisms located within the *IL2/IL21* chromosome region as well as within the *IL2RA* gene did not provide additional information (Tables 3 and 4, respectively).

**Table 3:** ***IL2/IL21 *allelic combinations (rs2069762, rs6822844 and rs907715) in uveitis patients and healthy controls from Spanish population**

**Allelic combination**	**Uveitis, n (%)**	**Controls, n (%)**	***P-*value**	**OR [95% CI]**
AGC	142 (39.3)	596 (39.2)	0.95	1.01 (0.79-1.28)
CGC	106 (29.3)	432 (28.4)	0.71	1.05 (0.81-1.36)
AGT	83 (22.9)	301 (19.8)	0.17	1.21 (0.91-1.61)
ATT	28 (7.8)	185 (12.2)	0.02	0.61 (0.39-0.94)

**Table 4:** ***IL2RA *allelic combinations (rs12722495, 2104286 and rs11594656) in uveitis patients and healthy controls from Spanish population**

**Allelic combination**	**Uveitis, n (%)**	**Controls, n (%)**	***P-*value**	**OR [95% CI]**
ATA	195 (50.8)	714 (47.0)	0.21	1.15 (0.92-1.45)
ATT	112 (29.1)	498 (32.6)	0.16	0.84 (0.65-1.08)
ACA	46 (12.0)	186 (12.4)	0.86	0.97 (0.68-1.39)
GCA	29 (7.5)	106 (7.0)	0.71	1.08 (0.69-1.69)

## Discussion

Although the etiology of uveitis is not fully understood yet, multiple evidences have strongly suggested that alterations in the immune system may underlie the dysfunction observed in endogenous uveitis [37]. To date, different functional studies have supported that the T-regulatory cells as well as Th17 cells seem to confer protection from and risk to intraocular inflammation, respectively [38]. IL-2 promotes development of regulatory T cells and confers protection from autoimmune disease [10] whereas IL-21 promotes differentiation of Th17 cells and is implicated in the development of several autoimmune diseases [15]. Interestingly, on the one hand IL-2 inhibits the differentiation of Th17 cells, although on the other hand it induces the expansion of Th17 cells once developed that mediate uveitis [13]. Therefore, taking into account all these evidences and the shared genetic component among the different autoimmune diseases, in the present study we aimed to analyze the influence of different polymorphisms located within the *IL2/IL21*, *IL2RA and IL2RB* loci, previously associated to multiple autoimmune disorders, on the non-anterior autoimmune uveitis susceptibility.

It is important to note that although we observed a weak association of the rs6822844 *IL2/IL21* polymorphism with uveitis, it did not reach statistical significance after multiple testing correction, probably due to the large number of tests performed and the limited statistical power to detect moderate effects (Additional file 1: Table S1). For this reason, further studies are needed in order to confirm the possible *IL2/IL21* genetic region influence on the non-anterior uveitis genetic background. This polymorphism has been studied in several autoimmune diseases such as celiac disease, type 1 diabetes and rheumatoid arthritis and showed a protective effect in all of them. In this way, our results for the rs6822844 are in agreement with the effect formerly observed for other conditions suggesting a common underlying mechanism [31,32,34]. The rs6822844 polymorphism probably may be acting as genetic marker of another nearby causal polymorphism in tight linkage disequilibrium with it. In addition, after confirmation of the *IL2/IL21* genetic region influence on uveitis genetic predisposition, more studies would be required in order to reveal the causal variant/s responsible and to clear up the influence of this region on the uveitis etiology. Importantly, a better knowledge of the uveitis etiology could allow a successful and more personalized treatment of the disease.

The association of *IL2RA* polymorphisms with autoimmunity is more complex and a given polymorphism may show the opposite effect in different autoimmune disorders [27]. Our results indicate that the analyzed *IL2RA and IL2RB* polymorphisms do not seem to play a significant role on the non-anterior uveitis genetic predisposition, similar to what has been reported in other autoimmune diseases such as inflammatory bowel disease and celiac disease. In these diseases underlying mechanisms different to the IL2-dependent pathway would probably be involved in the perturbed development of regulatory T cells. Recently, the *IL2RA* rs2104286 polymorphism has been associated with intermediate uveitis [39]. When we evaluated whether the studied genetic variants were involved in the different uveitis forms we did not observe the any association with the different uveitis subphenotypes. Nevertheless, this no evidence of association between the *Il2RA* locus and intermediate uveitis must be taken with caution because, although the statistical power of the overall analysis in the present study is high (Additional file 1: Table S1; 99% at the 5% significant level to identify the previously detected OR equal to 0.52), the statistical power of the stratified analysis for different uveitis forms is limited and, probably, moderate effects are hardly detected.

## Conclusions

Therefore, our results suggest that analyzed *IL2/IL21*, *IL2RA and IL2RB* polymorphisms do not seem to play a significant role on the non-anterior uveitis genetic predisposition although additional studies are needed to draw firm conclusions about the exact role of the analyzed genes in the susceptibility and clinical spectrum of uveitis.

## Competing interest

The authors declare that they have no competing interest.

## Authors’ contributions

MCC and AM participated in the design of the study, performed the genotyping, analyzed the data, and drafted the manuscript. JM made substantial contribution to the conception and design of the study, acquisition of data, coordination and drafting of the manuscript. MC-C, AF, AA, AM-B, VL, DD-V, RB, JC, DS, JL G-S, ER, MJR, MBG- E, JM M-V*,* NO-C participated in the design of the study, performed the statistical analysis and interpretation of data. All the authors revised critically the manuscript, gave necessary attention to ensure the integrity of the work presented, and approved the final version.

## Pre-publication history

The pre-publication history for this paper can be accessed here:

http://www.biomedcentral.com/1471-2350/14/52/prepub

## Supplementary Material

Additional file 1: Table S1 Overall statistical power for each analyzed genetic variant at the 5% significance level. Click here for file
