# Supplementary material for: Evaluation of the IL2/IL21, IL2RA and IL2RB genetic variants influence on the endogenous non-anterior uveitis genetic predisposition
Source: BMC Med Genet. 2013 May 15;14:52. doi: 10.1186/1471-2350-14-52 (PMC3658927; doi:10.1186/1471-2350-14-52)
Supplement: Additional file 1: Table S1 — Overall statistical power for each analyzed genetic variant at the 5% significance level. [file 1471-2350-14-52-S1.doc]

**Supplementary Table 1**. Overall statistical power for each analyzed genetic variant at the 5% significance level.

|  |  |  |  |  |  |
| --- | --- | --- | --- | --- | --- |
| **Overall uveitis** | | | | | |
| **SNP** | **OR=1.1** | **OR=1.2** | **OR=1.3** | **OR=1.4** | **OR=1.5** |
| ***IL2*** |  |  |  |  |  |
| rs2069762 | 12 | 31 | 59 | 80 | 92 |
| rs6822844 | 8 | 18 | 29 | 43 | 58 |
| rs907715 | 12 | 33 | 59 | 80 | 92 |
| ***IL2RA*** |  |  |  |  |  |
| rs12722495 | 7 | 13 | 29 | 43 | 58 |
| rs2104286 | 10 | 25 | 49 | 71 | 85 |
| rs11594656 | 12 | 33 | 58 | 79 | 92 |
| ***IL2RB*** |  |  |  |  |  |
| rs743777 | 12 | 33 | 60 | 81 | 93 |
